# Physiological and Metabolomic Responses of Kale to Combined Chilling and UV-A Treatment

**DOI:** 10.3390/ijms20194950

**Published:** 2019-10-08

**Authors:** Jin-Hui Lee, Min Cheol Kwon, Eun Sung Jung, Choong Hwan Lee, Myung-Min Oh

**Affiliations:** 1 Division of Animal, Horticultural and Food Science, Chungbuk National University, Cheongju 28644, Korea; jhjh@cbnu.ac.kr; 2 Brain Korea 21 Center for Bio-Resource Development, Chungbuk National University, Cheongju 28644, Korea; 3 Department of Bioscience and Biotechnology, Konkuk University, Seoul 05029, Korea; alscjf1102@naver.com (M.C.K.); chlee123@konkuk.ac.kr (C.H.L.); 4 Department of Systems Biotechnology, Konkuk University, Seoul 05029, Korea; jes708@naver.com; 5 Research Institute for Bioactive-Metabolome Network, Konkuk University, Seoul 05029, Korea

**Keywords:** photosynthetic rate 1, growth 2, stress 3, phenolic compound 4, chlorophyll fluorescence 5

## Abstract

Short-term abiotic stress treatment before harvest can enhance the quality of horticultural crops cultivated in controlled environments. Here, we investigated the effects of combined chilling and UV-A treatment on the accumulation of phenolic compounds in kale (*Brassica oleracea* var. *acephala*). Five-week-old plants were subjected to combined treatments (10 °C plus UV-A LED radiation at 30.3 W/m^2^) for 3-days, as well as single treatments (4 °C, 10 °C, or UV-A LED radiation). The growth parameters and photosynthetic rates of plants under the combined treatment were similar to those of the control, whereas UV-A treatment alone significantly increased these parameters. Maximum quantum yield (*Fv/Fm*) decreased and H_2_O_2_ increased in response to UV-A and combined treatments, implying that these treatments induced stress in kale. The total phenolic contents after 2- and 3-days of combined treatment and 1-day of recovery were 40%, 60%, and 50% higher than those of the control, respectively, and the phenylalanine ammonia-lyase activity also increased. Principal component analysis suggested that stress type and period determine the changes in secondary metabolites. Three days of combined stress treatment followed by 2-days of recovery increased the contents of quercetin derivatives. Therefore, combined chilling and UV-A treatment could improve the phenolic contents of leafy vegetables such as kale, without growth inhibition.

## 1. Introduction

Plants subjected to environmental stresses often generate excess amounts of reactive oxygen species (ROS), causing oxidative stress, which has a negative effect on plant growth and development. However, these responses depend on the concentrations of ROS generated, which are affected by the site of plant stress and duration of stress exposure [1]. When plants are subjected to excessive stress conditions, the balance between ROS and antioxidants that quench ROS is disrupted, thereby inducing programmed cell death or necrosis. However, when plants are exposed to more mild stress, ROS can act as signaling molecules that activate antioxidant defense mechanisms to help plants adapt to stressful environments [2]. This is a representative defense mechanism alongside other morphological and physiological mechanisms against abiotic stressors, with a detrimental effect on the survival of plants.

Short-term exposure to single chilling or UV treatment can improve plant tolerance to environmental stress [3,4,5]. When a plant is exposed to chilling, growth is inhibited due to a decrease in enzyme activity in the cell membrane. This reaction is caused by the excessive generation of ROS, such as hydrogen peroxide and oxygen free radicals, which is exacerbated by the imbalance between light absorption and light use due to the inhibition of the Calvin–Benson cycle activity [6]. Complex detoxifying defense mechanisms are induced in plants to protect them from oxidative damage. This process includes increases in the activities of antioxidant enzymes associated with the ascorbate/glutathione cycle, resulting in the removal of hydrogen peroxide from the cell compartments [7,8]. In addition, shielding components such as flavonoids accumulate in epidermal cells to prevent direct damage by UV irradiation, including damage to the photosynthetic apparatus and thylakoids in chloroplasts. Non-enzymatic antioxidants such as ascorbate (ASC), glutathione (GSH), carotenoids, and α-tocopherol also play important roles in plant responses to chilling. In particular, the structural chemistry of polyphenols plays a direct role in their activity as free radical scavengers [9]. The biosynthesis of phenolic compounds, such as hydroxycinnamic acids with antioxidant properties is activated by ROS generated by UV-induced limited CO_2_ assimilation; these phenolic compounds accumulate in glandular/secretory trichomes and adaxial epidermal cells [10]. Therefore, chilling and UV treatments during the cultivation period could represent excellent strategies for increasing the antioxidant contents of food crops [11,12].

The profiles of flavonoids, a representative antioxidant group closely involved in defense mechanisms against abiotic stress, vary in response to different stress factors. The biosynthesis of ortho-dihydroxy flavonoids was not induced by UV but was induced by chilling in *Arnica montana* [13]. In addition, phytochemical levels are sometimes enhanced when two different stressors are simultaneously applied to plants, pointing to their synergistic activities [14]. The flavonoid content was higher in bell pepper subjected to combined low-temperature and UV-B treatment versus single stress treatments [15]. The qualitative and quantitative synergistic effects of combined stress on phytochemical levels might result from complex signaling effects of cross defense mechanisms for single stressors. However, little is known about the biosynthesis of secondary metabolites in plants in response to complex environmental stress conditions.

We hypothesized that combined chilling and UV treatment would stimulate various secondary metabolite biosynthetic pathways, thereby, leading to the accumulation of various phytochemical compounds. Therefore, in this study, we set out to answer the following questions: (1) Do chilling and UV-A treatment act as stressors in kale to induce plant defense mechanisms? If so, how do these treatments affect plant growth? (2) Are different groups of secondary metabolites produced by different defense mechanisms in response to different types of stress? (3) How does combined chilling and UV treatment affect the production of secondary metabolites compared to single treatments?

## 2. Results

### 2.1. Chlorophyll Fluorescence

**Figure 1:**
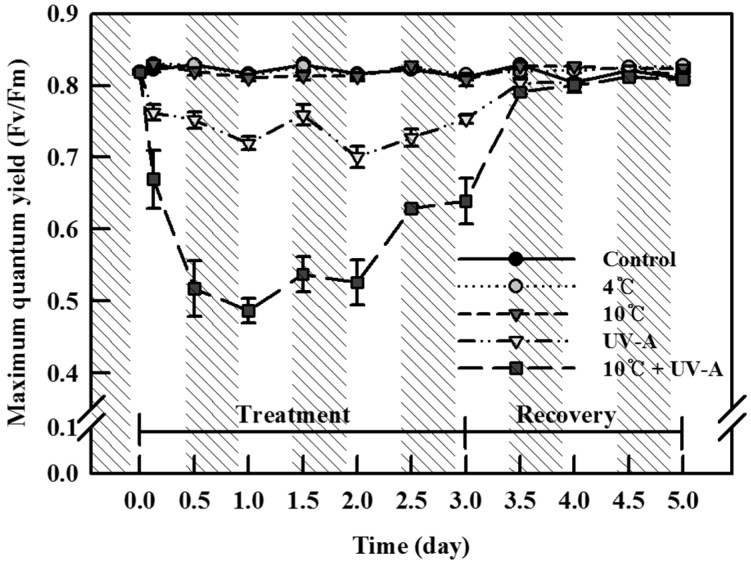
Maximum quantum yield (*Fv/Fm*) of kale leaves subjected to chilling (4 °C or 10 °C), UV-A, and combined stress treatments (10 °C + UV-A) for 3 days followed by 2 days of recovery. The vertical bars indicate standard errors (*n* = 9). Slashes indicate nighttime (0.5 day).

We measured changes of the maximum quantum yield (*Fv/Fm*) of photosynthesis as an indirect indicator of plant stress, as shown in Figure 1. No significant decrease in *Fv/Fm* occurred in response to the two single chilling treatments (4 °C and 10 °C) compared to the control. However, the *Fv/Fm* in plants treated by UV-A radiation (provided by LEDs) decreased after 3 h, remained at a lower level than the control until recovery (mean 0.74, ranging from 0.70 to 0.76), and was similar to the control value after recovery (mean 0.81). Combined chilling and UV-A treatment for 3 h led to a rapid decrease in *Fv/Fm*, with the lowest value maintained during the treatment period (mean 0.60, ranging from 0.49 to 0.66). When the plants were transferred to normal growth conditions, the value recovered rapidly, as was also observed after UV-A treatment (mean 0.81).

### 2.2. Growth Characteristics

**Figure 2:**
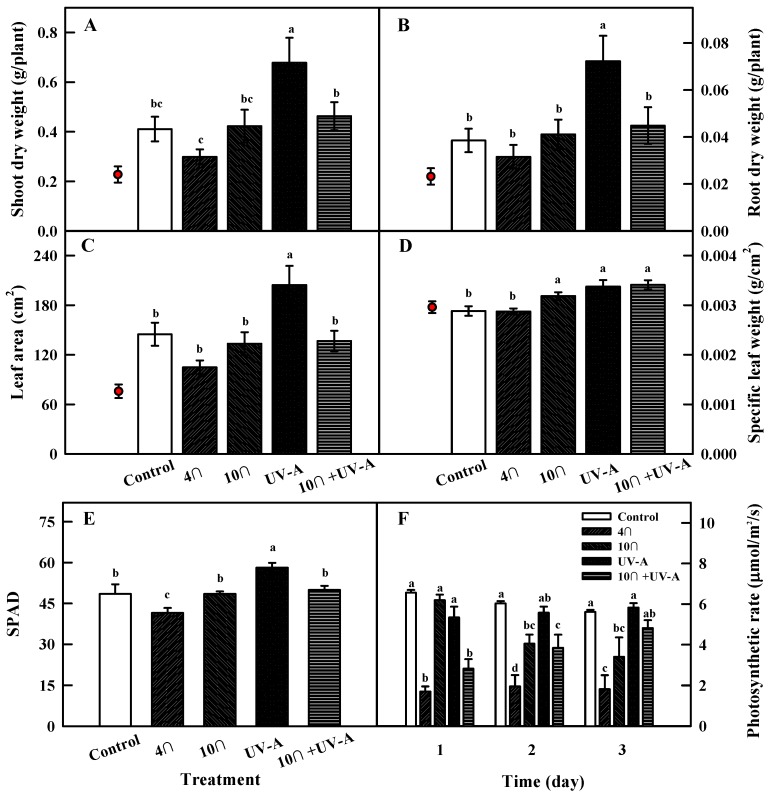
Shoot (**A**) and root (**B**) dry weight, leaf area (**C**), and specific leaf weight (**D**) of kale after 2 days of recovery from stress treatment. Red circles indicate each initial growth parameter immediately before treatment. SPAD value (**E**) of kale at 2 days of recovery and photosynthetic rate (**F**) of kale leaves subjected to chilling, UV-A, and combined treatments at 1 (28 h), 2 (52 h), and 3 days (76 h) of treatment. The vertical bars indicate standard errors (**A**, **B**, **C**, and **D**, *n* = 9; **E** and **F**, *n* = 4). Different letters above the bars indicate significant differences at *p*-value < 0.05.

Shoot and root growth significantly differed in kale plants subjected to 3 days of different stress treatments and 2 days of recovery (Figure 2A,B). Under 4 °C, the fresh weights of shoot and root appeared to decrease but did not significantly differ from control values. Plants subjected to UV-A treatment had the highest values; the fresh weights of shoot and root were 1.7 and 1.9 times higher than those of the control, respectively. By contrast, plants subjected to combined treatment maintained similar values to those of the control.

Similar results were obtained for leaf area measurements (Figure 2C), which did not significantly differ from the control in response to any treatment except UV-A, leading to a 1.4 times higher leaf area than the control.

Specific leaf weight (SLW; leaf thickness) showed a different pattern from the other growth parameters (Figure 2D). Chilling (10 °C), UV-A, and combined treatments led to significantly higher SLW than the control, especially combined treatment, which increased this value by approximately 18%, compared to the control.

### 2.3. Photosynthetic Rate

The SPAD values (chlorophyll content) and photosynthetic rates of kale at 2 days of recovery are shown in Figure 2. The SPAD value was significantly (19%) higher under UV-A, compared to the control, whereas the SPAD value under combined treatment was similar to the control. A temperature of 4 °C led to the lowest SPAD value (Figure 2E).

The photosynthetic rate changed in response to 1 to 3 days of single and combined treatments, with different patterns detected after different treatment periods (Figure 2F). The photosynthetic rates in the 4 °C and combined treatment were significantly lower than those of the other treatment groups and the control at 1 day of treatment. The photosynthetic rate at 10 °C began to decrease at 2 days of treatment and was 1.82 times lower at 3 days of treatment, versus the control. Plants subjected to 3 days of UV-A maintained similar photosynthetic rates to the control. Under combined treatment, the most rapid decrease in photosynthetic rate was observed at 1 day of treatment, but this value recovered with increasing treatment time. At 3 days of combined treatment, the photosynthetic rate was not significantly different from that of the control and the UV-A group.

### 2.4. Total Phenolic Content and Antioxidant Capacity

**Figure 3:**
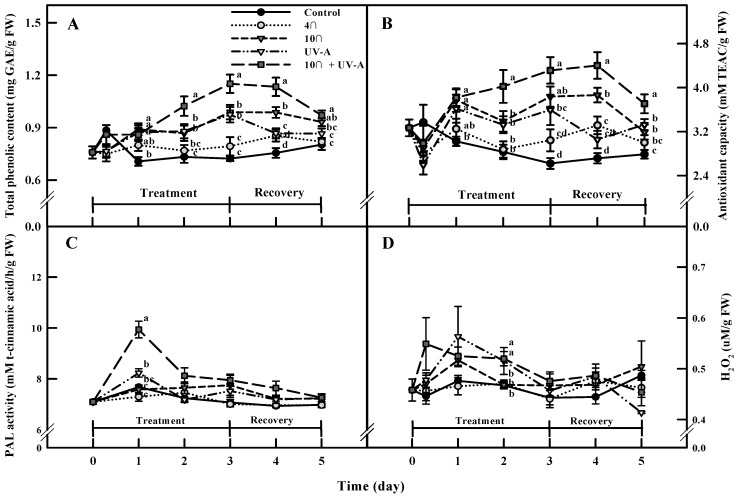
Total phenolic content (**A**), antioxidant capacity (**B**), phenylalanine ammonia-lyase (PAL) activity (**C**), and hydrogen peroxide levels (**D**) in kale subjected to chilling, UV-A, and combined stress treatments for 3 days, followed by 2 days of recovery. The vertical bars indicate standard errors (**A** and **B**, *n* = 9, **C** and **D**, *n* = 5). Different letters above the bars indicate significant differences at *p*-value < 0.05.

The total phenolic content and antioxidant capacity of plants subjected to single and combined treatments were significantly different during the treatment and recovery periods (Figure 3). The total phenol content of the control appeared to increase at 3 h after treatment, but the lowest value was detected at the beginning, during day 1 of treatment (Figure 3A). The total phenolic content significantly increased most strongly in response to 10 °C, followed by UV-A and combined treatment, as compared to the control at day 1 of treatment. The increase reached a peak after 3 days of treatment and 1 day of recovery. The total phenolic content increased 1.59- and 1.50-fold after 3 days of combined treatment and after 1 day of recovery, respectively, compared to the control. Finally, the total phenolic content increased significantly under 10 °C (1.36- and 1.30-fold after 3 days of 10 °C treatment and 1 day of recovery, respectively).

The changes in the antioxidant capacity were similar to the changes in the total phenol content (Figure 3B). There were significant increases in antioxidant capacity in response to both single and combined stress, compared to the control. The most significant increase was observed in response to 1 day of combined treatments (1.25-fold increase) and after 3 days of combined treatment and 1 day of recovery (1.64- and 1.62-fold increases, respectively; Figure 3B).

### 2.5. Phenylalanine Ammonia-Lyase (PAL) Activity and ROS (H_2_O_2_) Levels

PAL activity increased by 7% and 29% in response to 1 day of UV-A and combined treatment, respectively. PAL activity was maintained at a relatively high level in response to combined treatment, until 1 day after recovery (Figure 3C).

H_2_O_2_ content showed dynamic fluctuations in response to single and combined stress during the entire treatment period (Figure 3D). H_2_O_2_ content increased rapidly compared to the control, 3 h after the combined and other treatments. Plants subjected to all treatments except chilling stress (4 °C) had higher H_2_O_2_ contents than the control at day 1 of treatment. H_2_O_2_ contents in response to both combined and UV-A treatments gradually decreased 2 days after treatment but were significantly higher than the control (11% and 10%, respectively). However, there was no significant difference in H_2_O_2_ content after 2 days of 4 °C or 10 °C treatment, compared to the control.

### 2.6. Nontargeted Metabolite Profiling

**Figure 4:**
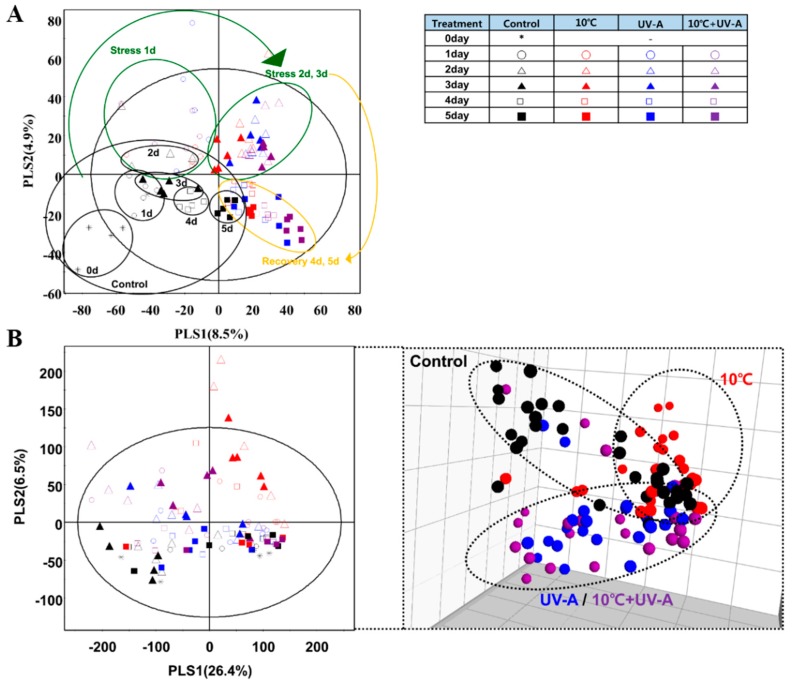
Partial least square–discriminant analysis (PLS–DA) score plots derived from ultra-performance liquid chromatography–quadrupole–time-of-flight–mass spectrometry (UPLC–Q–TOF–MS) (**A**) and gas chromatography–time-of-flight–mass spectrometry (GC–TOF–MS) (**B**) data for kale leaves subjected to 10 °C, UV-A, and combined treatments for 3 days of stress treatment and 2 days of recovery.

To investigate the effects of temperature or light stress on metabolite levels in kale leaves, we performed nontargeted metabolite profiling by ultra-performance liquid chromatography–quadrupole–time-of-flight–mass spectrometry (UPLC–Q–TOF–MS) and gas chromatography–time-of-flight–mass spectrometry (GC–TOF–MS) followed by multivariate statistical analysis. Principal component analysis (PCA) based on the results of UPLC–Q–TOF–MS analysis clearly discriminated the control group from the stress-treated groups by PC1 (8.6%), but no clear separations were observed among the stress-treated groups or treatment days (Supplementary Figure S1A). In GC–TOF–MS-derived PCA score plots, the control and 10 °C groups were clearly discriminated by PC1 (26.8%) and PC2 (26.5%). The UV-A and 10 °C + UV-A groups were not separated from each other (Supplementary Figure S1B). Partial least square-discriminant analysis (PLS–DA) revealed clearer grouping patterns based on stress treatments and days (Figure 4). The UPLC–Q–TOF–MS-derived PLS–DA model revealed steady changes in secondary metabolite levels in leaves during the stress treatments and recovery period. Compared to the control, members of the stress-treated groups were widely dispersed according to treatment days, although some patterns were observed in each stress-treated group within treatment dates. The PLS–DA of GC–TOF–MS data sets grouped members based on the stress type rather than the date of stress treatment.

**Figure 5:**
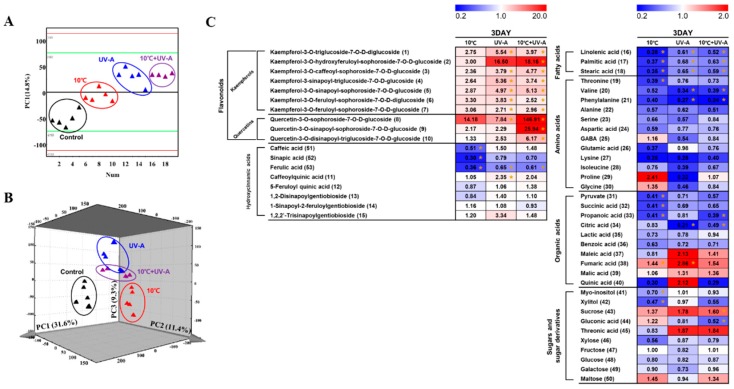
PCA score plots derived from UPLC–Q–TOF–MS (**A**) and GC–TOF–MS (**B**) data for kale leaves after 3 days of 10 °C, UV-A, and combined treatments. (**C**) Heat map representation of the relative abundance of significantly discriminant metabolites (VIP > 0.7) based on the orthogonal partial least squares-discriminant analysis (OPLS–DA) model (Supplementary Figure S2). The values represent the fold change with respect to the control. Asterisks indicate significant differences (*p*-value < 0.05) between the control and each treatment.

To identify metabolic changes in kale leaves in response to 10 °C, UV-A, and combined treatments, we performed multivariate statistical analysis on samples stressed for 3 days (the longest stress treatment; Figure 5). In the PCA score plots of the UPLC–Q–TOF–MS data sets, the UV-A and combined treatment groups were clearly separated from the control, according to PC1 (14.8%) but not from the 10 °C group. However, in the GC–TOF–MS-derived PCA score plot, all stress-treated groups were clearly discriminated from the control groups, but the UV-A and combined treatment groups were very close to each other. Finally, we performed orthogonal partial least squares-discriminant analysis (OPLS–DA) of the control group versus each stress-treated groups to identify the different metabolites between groups (Supplementary Figure S2), which were selected on the basis of the variable importance in projection (VIP) > 0.7 values of each OPLS–DA model. Fifty-three significantly discriminant metabolites were selected for further analysis, including flavonoids, hydroxycinnamic acids, fatty acids, amino acids, organic acids, sugars, and sugar derivatives (Supplementary Tables S1 and S2).

### 2.7. Metabolic Variations at 3 Days of Treatment

We visualized the relative levels of discriminant metabolites (VIP > 0.7) in kale leaves between stress-treated groups using a heat map (Figure 5C). The overall patterns of changes in primary and secondary metabolite levels in the UV-A and combined treatments were quite similar. Among flavonoids, the levels of kaempferol glycosides and quercetin glycosides appeared to increase in all stress-treated groups (10 °C, UV-A, and combined treatment) compared to the control. However, the increases in kaempferol glycoside contents were statistically significant only in the UV-A and the combined treatment. The levels of quercetin glycosides markedly increased only in the combined treatment. Hydroxycinnamic acid levels were also affected by stress treatment. In particular, ferulic acid (53) levels were significantly reduced in all stress-treated groups compared to the control. In addition, caffeic acid (51) and sinapic acid (52) levels were significantly reduced in the 10 °C treatment.

The fluctuations in primary metabolite levels, including fatty acids, amino acids, organic acids, and sugars, were similar among the treatment groups compared to the control. However, notable differences were observed in the 10 °C groups, with more significant decreases in the levels of several metabolites than the other groups, such as linolenic acid (16), palmitic acid (17), stearic acid (18), threonine (19), pyruvate (31), succinic acid (32), myo-inositol (41), and xylitol (42). The relative levels of phenylalanine, alanine, citric acid, linolenic acid, palmitic acid, and stearic acid were significantly reduced in both the UV-A and combined treatment, with similar fold changes. Propanoic acid (33), sucrose (43), and gluconic acid (44) levels were significantly altered only in the combined treatment. We constructed S-plots of each OPLS-DA model to identify the metabolites with significant contributions to the maximum disparity between the control and stress-treated groups (Supplementary Figure S2). The axis represents the predictive components for covariance.

To evaluate the correlations between metabolites and antioxidant activities, we performed correlation analysis between significantly discriminate metabolites and bio-activities (Supplementary Figure S3). Among these, most flavonoids and hydroxycinnamic acids showed a positive correlation with bio-activities. Among the primary metabolites were several organic acids and sugars [pyruvate (31), succinic acid (32), maleic acid (37), fumaric acid (38), malic acid (39), myo-inositol (41), sucrose (43), threonic acid (45), fructose (47), glucose (48), galactose (49), and maltose (50)].

### 2.8. Changes in Secondary Metabolite Levels after 2 Days of Recovery

**Figure 6:**
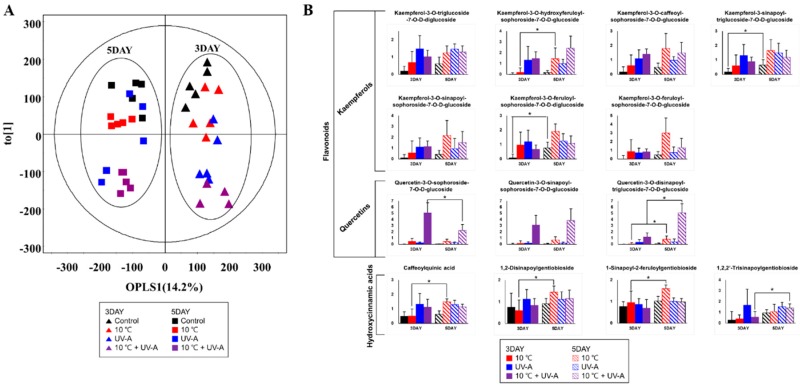
OPLS–DA score plots based on UPLC–Q–TOF–MS data from day 3 (after 3 days of stress treatment) versus day 5 (after 2 days of recovery) (**A**). Bar graphs of the relative levels of secondary metabolites (variable importance in projection (VIP) > 0.7) on days 3 and 5 (**B**). Solid bars—day 3, striped bars—day 5, black bars—control, red bars—10 °C, blue bars—UV-A, purple bars—combined treatment. Asterisks indicate significant differences (*p*-value < 0.05) between days 3 and 5.

To detect changes in secondary metabolite levels after 2 days of recovery, we performed OPLS–DA of samples at the 3- and 5-day time-points (Figure 6). We selected significantly discriminated metabolites according to the VIP value (>0.7) and visualized the relative levels in a bar graph. According to graph, most of flavonoids and hydroxycinnamic acid levels showed no significant changes on day 5 in the UV-A and the combined treatment groups, compared to day 3. However, the levels of these metabolites increased in the 10 °C after 2 days of recovery. Specifically, kaempferol-3-O-hydroxyferuloyl-sophoroside-7-O-D-glucoside (2), quercetin-3-O-disinapoyl-triglucoside-7-O-D-glucoside (10), caffeoylquinic acid (11), and 1,2,-disinapoylgentiobioside (13) levels significantly increased during this period. The levels of several metabolites in the combined treatment group also increased slightly during recovery, and a significant increase in quercetin-3-O-disinapoyl-triglucoside-7-O-d-glucoside (10) levels was also observed.

## 3. Discussion

### 3.1. Effects of Single and combined Stress Treatments on Plant Biomass

The growth of kale plants subjected to 3 days of low-temperature, varied according to the temperatures. Plant growth was not inhibited at 10 °C, but shoot dry weight and root fresh weight were significantly lower at 4 °C, compared to the control. Inhibited plant growth at low-temperature results from a decrease in the photosynthetic rate due to the suppression of enzyme activity in the cell membrane [16]. The photosynthetic rate significantly decreased at 1, 2, and 3 days of 4 °C treatment compared to the control (Figure 2F). The significant decrease in the SPAD value detected at 4 °C (Figure 2E) could result from the structural destruction and degradation of photosynthetic pigments [17]. However, the photosynthetic rate at 10 °C was similar to that of the control at day 1 of the treatment but gradually decreased after 2 and 3 days of treatment, perhaps reflecting the cumulative effects of physiological disorders due to the chilling stress [18]. Although 10 °C reduced the photosynthetic rate, no decrease in growth was observed. Perhaps the benign low-temperature of 10 °C activated a defensive mechanism in kale, a cold-tolerant crop, and this short-term treatment had a minimal effect on reducing the carbon assimilation rate.

Two days of recovery following 3 days of UV-A irradiation had a pronounced effect on biomass accumulation (Figure 2). Verdaguer et al. [19] reported that UV-A radiation was absorbed directly into light-harvesting pigments such as chlorophylls and carotenoids; therefore, UV-A could promote plant growth under low-light intensities that did not reach the saturation point. In our previous study, the shoot fresh-weight of kale subjected to UV-A LED light with 370- and 385-nm peak wavelengths increased by 32% and 58%, respectively, when cultivated under a light intensity below the light saturation point [20]. In this study, it is likely that UV-A radiation contributed to the photosynthetic photon flux density (PPFD) by functioning as a supplemental light source and enhanced plant growth, as the PPFD supplied by plant growth light was 132 μmol m^−2^ s^−1^, which was far below the light saturation point. The SPAD values were also significantly higher under UV-A treatment versus the control (Figure 2E), which was consistent with the finding that UV-A sources had a positive effect on photosynthesis by helping plants produce chlorophylls [19]. However, the photosynthetic rate under UV-A LED was not significantly different from that of the control after 3 days of treatment, suggesting that UV-A might have a positive effect on other aspects of plant growth besides the photosynthetic rate. Indeed, UV-A treatment promoted the uptake of nitrogen compounds (NO_2_^−^ and NH_4_^+^) and stimulated the activity of nitrate reductase and nitrite reductase in the nitrogen metabolic pathway in radish plants, resulting in growth promotion [21]. More research is needed to clarify the reasons for the increased growth in kale in response to UV-A LED radiation.

Applying 10 °C and UV-A treatment at the same time did not inhibit plant growth, compared to the control (Figure 2). As mentioned above, these results suggest that UV-A promotes plant growth, even at 10 °C, and that the decrease in growth due to the chilling treatment does not offset this effect. The photosynthetic rates significantly decreased at day 1 of a combined treatment compared to the control, but this value tended to recover with treatment time (Figure 2F). Photosystem I (PS I) was the primary site of photoinhibition in plants under chilling stress in the light [22]. UV radiation before and during chilling stress deactivated the oxygen-evolving complex. Therefore, under this treatment, photoinhibition was mitigated by reduced electron transport to PS I, and the recovery of CO_2_ assimilation was accelerated [22].

### 3.2. Effects of Single and Combined Stress Treatments on the Biosynthesis of Bioactive Compounds

In general, the daily rhythms of low molecular weight antioxidant levels and antioxidative enzyme activities are induced by endogenous circadian and exogenous variables [23]. In the control, we detected rapid increases in the total phenolic content and antioxidant capacity after 3 h of treatment, likely due to the cyclic nature of these phytochemicals. Therefore, we sampled leaf tissue at the same time of day from 1 to 5 days of treatment to detect changes resulting from stress treatments.

Plant secondary metabolite biosynthesis was activated as a defense mechanism against biotic and abiotic stress [3,4]. Indeed, the total phenolic content and the antioxidant activity significantly increased under all treatments (Figure 3A,B). Even with the same treatment periods, 10 °C more effectively increased the total phenolic content and the antioxidant capacity than a 4 °C treatment, indicating that the accumulation of bioactive compounds and the degree of physiological disorders vary, depending on the precise temperature. Indeed, the distribution of carbon sources to secondary metabolite biosynthesis decreased at 4 °C, due to a significant decrease in the photosynthesis rate, and enhanced production of ROS such as H_2_O_2_ occurred at 10 °C during early treatment. Pastori et al. [24] showed that H_2_O_2_ concentrations increased in 15 °C-treated maize after subsequent exposure to 20 °C, 18 °C, and 15 °C, which stimulated the generation of superoxide dismutase, GSH, and ASC. In this study, the highest total phenolic content and antioxidant capacity were detected at both day 1 of recovery and after 3 days of treatment. This increase in the bioactive compound levels after recovery could be due to the regeneration of active forms of antioxidants [25].

UV-A also induced significant increases in bioactive compound levels during both the treatment and recovery periods. In particular, the total phenolic content significantly increased (by 34% versus the control) at 3 days of UV-A treatment (Figure 3A). Similarly, treatment with solar UV-A radiation using UV cutoff filters and UV-A lamps increased the phenolic and antioxidant contents in birch and lettuce [26,27], and treatment with several UV-A LEDs with specific peak wavelengths increased antioxidant, total phenolic, and anthocyanin contents in basil and pak choi [28]. These increases were caused by the increased synthesis of UV-absorbing compounds, such as flavonoids on the leaf epidermis, which function as shielding components to protect the photosynthetic apparatus centering on photosystem II (PS II) from UV-A damage or the accumulation of antiradical compounds to quench ROS generated by UV-A stress [10,29]. In this study, we detected a decrease in *Fv/Fm* during UV-A treatment, reflecting an abnormality in the electron transfer in PS II, supporting the notion that UV-A radiation functions as a stressor that increases the bioactive compound levels as part of a defense mechanism against stress. In addition, the rapid increase in H_2_O_2_ levels at day 1 of UV-A supports the notion that ROS act as signaling molecules to induce the biosynthesis of phytochemicals.

Interestingly, both total phenol and antioxidant contents were highest in plants under combined 10 °C and UV-A treatment (Figure 3A,B), indicating that these two stressors have synergetic effects on plants when applied simultaneously. The combined treatment showed the highest H_2_O_2_ content and lowest *Fv/Fm* value at day 1 of treatment, which are signs of plant stress. However, unlike plants under the two chilling treatments, the photosynthetic rate of plants under the combined treatment gradually recovered, indicating that the carbon source could readily be distributed to secondary metabolite pathways. *Salix myrsinifolia* subjected to combined treatment with UV-A radiation and high-temperature showed significant increases in the levels of phenolic acids and dicoumaryl derivative [30], and combined low-temperature and UV-B radiation treatment increased the flavonoid content more strongly than other single stress in bell pepper [15]. Cold (9 °C) increased the accumulation of UV-A and UV-B screening pigments more strongly than higher temperature treatments (12 °C, 15 °C, 18 °C, and 21 °C) in *Vicia faba* [31]. In general, UV-damaged DNA was restored by DNA photolyase and through the nucleotide excision repair processes [32]. The enzymatic recovery of photochemical damage was inhibited at low-temperatures, resulting in an imbalance in DNA damage and recovery. Plants resolve this imbalance by enhancing the accumulation of screening pigments [31].

### 3.3. Effects of Single and Combination Stress Treatments on Metabolite Levels

We performed non-targeted metabolomics to uncover the differences in metabolic responses to light or low-temperature stress. Secondary metabolites play important roles in plant adaptation and environmental responses [33]. Treatment with 3 days of UV light or low-temperature stress, induced marked changes in thee metabolite levels in kale leaves (Figure 4 and Figure 5). In particular, flavonoid levels were higher in all stress-treated groups, compared to the control. However, no significant differences in flavonoid levels were detected between the 10 °C and the control, but UV-A and combined treatments led to significant increases in the levels of these compounds. We previously determined that 3 days of 5 °C resulted in a significant accumulation of antioxidant phenolics in kale [11]. Since kale is a cold-tolerant plant [34], perhaps 10 °C for 3 days is too mild to induce flavonoid accumulation.

Similar levels of kaempferol glycosides were detected in both the UV-A and combined treatment, whereas quercetin glycoside levels dramatically increased (more than 10-fold) in the combined treatment compared to the 10 °C and UV-A groups. In addition, the combined treatment showed the highest total phenolic content and antioxidant activity among groups (Figure 3A,B). The 10 °C and UV-A groups also showed significantly higher antioxidant activities than the control, but there was no significant difference between the treatment groups. Antioxidant activity was positively correlated with flavonoid levels in kale leaves. Quercetin glycosides showed higher ROS scavenging activity than kaempferol glycosides [35]. In addition, quercetin-3-O-sophoroside-7-O-D-glucoside, quercetin-3-O-sinapoyl-sophoroside-7-O-D-glucoside, and quercetin-3-O-disinapoyl-triglucoside-7-O-D-glucoside showed different antioxidant activities (1.89, 1.57, and 4.19, respectively) [36]. Therefore, perhaps the high quercetin glycoside contents in plants in the combined treatment contribute to the higher antioxidant activity in this group compared to the single treatments. The increased accumulation of these compounds might be due to the synergistic effects of 10 °C and UV-A treatments.

We detected notable decreases in the levels of hydroxycinnamic acids, including caffeic acid, ferulic acid, and sinapic acid, in the 10 °C at 3 days of stress treatment (Figure 5C), which might be related to lignification and suberin deposition. Lignin and suberin are complex polymers that form from a mixture of simple phenylpropanoids, such as caffeic, ferulic, and sinapic acids [37]. In general, cold stress induces the accumulation of phenolic compounds, which are subsequently incorporated into the cell wall as lignin or suberin [38]. Such cell wall thickenings are not only more resistant to pathogen attack, but they might also protect the plant from freezing stress [38]. Therefore, perhaps kale leaves exposed to 10 °C for 3 days exhibited changes in metabolism to overcome cold stress.

We also observed changes in primary metabolite levels after 3 days of 10 °C, UV-A, and combined treatments (Figure 4 and Figure 5), particularly for fatty acids and most amino acids. Plants exposed to abiotic stress often exhibit reduced photosynthesis or respiration, leading to energy starvation and retarded growth [39]. Plants under energy starvation produce energy through the oxidation of amino acids and the degradation of fatty acids and starch, until photosynthesis is completely recovered [40]. Therefore, the decrease in amino acid and fatty acid levels in kale might be associated with energy generation under stress conditions. In the 10 °C group, we detected notable differences in the levels of several amino acids, including proline, glycine, and threonine, compared to other treatment groups (Figure 5C). Proline accumulates in plants, in response to various environmental stresses and plays an important role as an osmolyte and chemical chaperone [40]. In addition, in response to abiotic stress and energy shortage, threonine is converted into isoleucine and catabolized into intermediates of the TCA cycle for energy production [41].

We detected significant decreases in the phenylalanine levels in both the UV-A and combined treatment groups. Phenylalanine levels also appeared to decline in the 10 °C, but this decrease was not significant. These results were similar to the patterns of flavonoid accumulation in the stress treatment (Figure 5). Phenylalanine is an entry compound of the phenylpropanoid biosynthesis pathway, which leads to the biosynthesis of flavonoids in plants [41]. All phenylpropanoids are derived from cinnamic acid, which is formed from phenylalanine, by the action of phenylalanine ammonia-lyase (PAL), the branch point enzyme between primary and secondary (phenylpropanoid) metabolism [42]. In this study, PAL activity significantly increased in the stress treatment groups compared to control in the order of the combined treatment > UV-A > 10 °C (Figure 3C), which was consistent with the variation in phenylalanine and flavonoid levels.

Finally, we investigated the metabolite changes in kale leaves in response to 2 days of recovery and detected notable changes in the flavonoid levels (Figure 6). The levels of most flavonoids decreased after 2 days of recovery in the UV-A and the combined treatment groups, but they increased in the 10 °C group. In a previous study of grape leaves, the contents of total phenolics and some flavonoids decreased in response to cold stress but increased after recovery, perhaps due to the increased biosynthesis of antioxidative compounds during recovery [43]. In this study, flavonoid levels showed a mild increase in the 10 °C group after 3 days of stress but increased further after the recovery period. These observations suggest that treatment with low-temperatures (such as 10 °C) could lead to the increased accumulation of flavonoids in kale after a recovery period, unlike other treatments. However, further studies are needed to confirm these metabolic changes through the analysis of flavonoid-related gene expression.

## 4. Materials and Methods

### 4.1. Plant Materials and Cultivation Conditions

Kale (*Brassica oleracea* var. *acephala*) seeds (Asia Seed Co., Ltd. Seoul, Korea) were sown in seed growth packs (Seed Pack, Useem Instruments Inc., Suwon, Korea) in a growth chamber (air temperature 20 °C, relative humidity 60%, CO_2_ 400 μmol mol^−1^, light period 12 h, PPFD 132 ± 5 µmol m^−2^ s^−1^) under fluorescent lamps, for 2 weeks, planted in a deep flow system, and cultivated for 3 weeks. Half-strength Hoagland and Arnon nutrient solution [44] was used, with the electrical conductivity (EC) and pH adjusted to 1.2 dS m^−1^ and 6.0, respectively, using a digital multiparameter meter (Multi 3430; WTW, Weilheim, Germany), once every 3 days. The solution was renewed every 2 weeks to reduce the effects of the altered nutrient composition on kale.

### 4.2. Environmental Stress Treatments

Seedlings at 3 weeks after transplantation were subjected to the following treatments: (1) 4 °C, (2) 10 °C, (3) UV-A provided by LEDs at 30 W/m^2^ (peak wavelength; 370 + 385 nm), and (4) 10 °C plus UV-A provided by LEDs at 30 W/m^2^. Four stress treatments were applied to the plants for 3 days, which were subsequently allowed to recover for 2 days under normal growth conditions. UV-A radiation was continuously provided by LEDs for 72 h.

Comparable conditions were used for the control (20 °C without UV-A radiation). The temperature was measured with a portable sensor in the growth chamber with a fluctuation range of ±1.8 °C. The UV energy of the plant canopy top was determined with a spectroradiometer (JAZ spectrometer, Ocean Optics, Dunedin, FL, USA).

### 4.3. Chlorophyll Fluorescence Measurements

Chlorophyll fluorescence was measured to assess the potential quantum yield of PS II (*Fv/Fm*), which indicates the stress level of plants subjected to environmental stress conditions [45]. *Fv/Fm* values were determined with the same leaves used to determine the photosynthetic rate, after 30 min of dark adaptation, using a chlorophyll fluorescence meter (PAM 2000; Heinz Walz GmbH, Effeltrich, Germany). Data were collected from plants every 12 h during the treatments. The saturating light pulse was 20 kHz, and radiation was performed at 1100 μmol/m^2^/s PPFD.

### 4.4. Growth Characteristics

The growth parameters of kale leaves, such as root and shoot weights (dry and fresh), leaf area, SLW, and SPAD value, were determined immediately before treatment and after 2 days of recovery. After measuring the fresh-weights of shoots and roots using an electronic scale (SI-234; Denver Instruments, Denver, CO, USA), the shoots and roots were dried in a 70 °C oven for 3 days, before measuring the dry weights. Leaf area was measured using an area meter (LI-3100; LI-COR, Lincoln, NE, USA), and SLW was calculated by dividing the leaf dry weight by the leaf area.

### 4.5. Chlorophyll Content and Photosynthetic Rate Measurements

SPAD value was measured with a portable chlorophyll meter (SPAD-502, Minolta Camera Co. Ltd., Osaka, Japan) to indirectly calculate the chlorophyll contents of the leaves.

Photosynthetic rate measurements were conducted on the third fully expanded leaves from top, using a portable photosynthesis system (LI-6400; Li-COR, Lincoln, NE, USA) at 1, 2, and 3 days of treatment. When measuring photosynthetic rates, the temperature, CO_2_ concentration, and flow rate inside the leaf cuvette were set to 20 °C, 400 μmol/mol, and 350 μmol/s, respectively.

### 4.6. Total Phenolic Content and Antioxidant Capacity

Fresh leaf tissue (approximately 0.2 g) was collected immediately before treatment and at 1-day intervals during the entire experimental period. The samples were stored at −70 °C prior to the analysis of the total phenolic content and the antioxidant capacity. The total phenolic content and antioxidant capacity were measured by grinding frozen plant tissue samples in liquid nitrogen and 3 mL 80% acetone, using a mortar and pestle. The total phenolic content and antioxidant capacity were analyzed using the Folin–Ciocalteu method [46] and the 2,2′-azino-bis method (3-ethylbenzothiazoline-6-sulfonic acid) (ABTS; Sigma-Aldrich, St. Louis, MO, USA) [47], with minor modifications. Total phenolic content and antioxidant capacity were expressed as gallic acid equivalent (mg) per fresh-weight (g) (GAE mg/g FW) and trolox-equivalent antioxidant capacity (mM) per fresh-weight (g) (TEAC mM/g FW), respectively.

### 4.7. ROS (H_2_O_2_) Measurements

H_2_O_2_ content was analyzed using an EZ-hydrogen peroxide assay kit (Oxidative Stress Assay kit; DoGenBio Co., Ltd., Seoul, Korea), according to the manufacturer’s instructions. In brief, fresh leaf tissue (approximately 0.1 g) was collected and stored in a deep freezer (DF8524; IlShinBioBase, Dongducheon, Korea) at −70 °C until analysis. The frozen sample was ground to a fine powder with a mortar and pestle in liquid nitrogen and homogenized in 2 mL of 100 mM potassium phosphate buffer (pH = 7.5). The extract was centrifuged at 18,000× *g* for 20 min, and the supernatant was filtered through a 0.22-µm syringe filter. The sample (50 μL) was transferred to a 96-well plate, and 50 μL of working solution was added to each well. The solution was incubated for 30 min in the dark, and absorbance was recorded at 560 nm, using an ELISA plate reader (Epoch; BioTek, Winooski, VT, USA). A standard curve was constructed for the known concentrations of H_2_O_2_ (10, 5, 2.5, 1.25, 0.625, 0.3125, and 0.15625 μM).

### 4.8. PAL Activity Measurements

PAL activity was determined as described by Boo et al. [48], with minor modifications. Approximately 0.5 g of the fresh leaf tissue was collected at 1-day intervals over the course of 5 days (3 days of treatment followed by 2 days of recovery) and stored in a deep freezer (DF8524; IlShinBioBase, Dongducheon, Korea) at −70 °C until analysis. Leaf samples were ground to a powder in a ceramic mortar in liquid nitrogen. The samples were extracted in a mixture of 10 mL of 25 mM borate buffer (pH 8.8) and 2 mL of 3 mM β-mercaptoethanol (Sigma-Aldrich, St. Louis, MO, USA). The extracts were centrifuged at 900× *g* for 20 min. A 0.5-mL aliquot of the supernatant was mixed with 2.5 mL of 25 mM borate buffer (pH 8.8) and 2.5 mL of 10 mM L-phenylalanine (Sigma-Aldrich, St. Louis, MO, USA) at 40 °C, for 2 h. The reactions were terminated by the addition of 100 μL of 5 N HCl, and the absorbance was measured against a blank at 290 nm with a spectrophotometer (UV-1800; Shimadzu, Kyoto, Japan). Enzyme activity was expressed as millimolar of trans-cinnamic acid per hour per gram of fresh weight (mM trans-cinnamic acid/h/g FW).

### 4.9. Metabolite Extraction

Each sample was lyophilized for 72 h and ground to a fine powder. The samples (15 mg) were extracted with 1 mL of 70% aqueous methanol, using a MM400 mixer mill (Retsch^®^; Haan, Germany) at a frequency of 30 s^−1^ for 10 min and sonication for 5 min at 4 °C (Hettich Zentrifugen Universal 320, Tuttlingen, Germany). The extracts were centrifuged at 13,000 rpm for 10 min at 4 °C. The supernatants were filtered through a 0.2-μm polytetrafluoroethylene syringe filter and thoroughly dried in a speed-vacuum concentrator (Biotron, Seoul, Korea). The dried samples were redissolved in 70% aqueous methanol prior to analysis.

### 4.10. UPLC–Q–TOF–MS and UHPLC–LTQ–IT–MS/MS Analysis

UPLC–Q–TOF–MS and UHPLC–LTQ–IT–MS/MS were performed as described by Park et al. [49]. To obtain reliable MS data, quality control (QC) samples comprising pooled mixed samples (5 μL) were analyzed after every 10 samples.

### 4.11. GC–TOF–MS Analysis

For the GC–TOF–MS analysis, each dried sample was subjected to two derivatization steps (oximation and silylation). The derivatization steps were performed by incubating the extracts with 40 μL of methoxyamine hydrochloride in pyridine (20 mg/mL) at 30 °C for 90 min. After adding 40 μL of *N*-methyl-*N*-trimethylsilyl trifluoroacetamide (MSTFA), the reaction mixture was incubated for 30 min at 37 °C. The derivatized samples were injected into the GC–TOF–MS system and analyzed as described by Park et al. [49]. To obtain reliable MS data, the QC samples were analyzed after every 10 samples.

### 4.12. Statistical Analysis

Statistical analyses of all data except metabolite data were conducted using the SAS software (SAS 9.2; SAS Institute, Cary, NC, USA). The mean and standard error (±SE) of each treatment are shown. Statistical analysis was carried out via analysis of variance (ANOVA). The mean values of all treatments were compared using Duncan’s multiple range test at *p* < 0.05.

GC–TOF–MS and UPLC–Q–TOF–MS raw data sets and multivariate statistical analyses were performed as described by Jung et al. [50]. The variables were selected on the basis of the VIP values, and significance was tested by ANOVA and Duncan’s multiple-range tests, using the PASW Statistics 18 software (SPSS, Inc., Chicago, IL, USA). The heat map and correlation map were visualized using the MEV software (version 4.8; Cluj-Napoca, Romania).

## 5. Conclusions

UV-A irradiation significantly increased plant growth parameters in kale as compared to the control. Although chilling stress inhibited plant growth, plants under combined cold–UV-A stress maintained similar growth parameters. Treatment with chilling plus UV-A radiation increased ROS levels in kale, which stimulated the biosynthetic pathways of secondary metabolites (PAL activity) and induced the accumulation of phenolic antioxidant compounds. In addition, the secondary metabolite profiles varied, depending on stress factors such as low-temperature and UV-A radiation, with synergistic effects detected under combined treatment. Therefore, treatment with the proper level of combined chilling and UV-A treatment could improve the phenolic contents of kale plants and it would be a potential technology for improving the nutritional quality of vegetables, as well as medicinal plants cultivated in controlled environments, such as plant factories and vertical farms. Additional studies focusing on changes in the secondary metabolic pathways in response to different abiotic stress treatments should be conducted to establish methods for enhancing the levels of bioactive compounds in crops in the future.

## Supplementary Materials

Supplementary materials can be found at https://www.mdpi.com/1422-0067/20/19/4950/s1.

## Abbreviations

ROS	Reactive oxygen species
ASC	Ascorbate
GSH	Glutathione
SLW	Specific leaf weight
UPLC-Q-TOF-MS	Ultra-performance liquid chromatography-quadrupole-time of flight mass spectrometry
GC-TOF-MS	Gas chromatography time-of-flight mass spectrometry
PCA	Principal component analysis
PLS-DA	Partial least square-discriminant analysis
VIP	Variable importance in projection
PPFD	Photosynthetic photon flux density
PSI	Photosystem I
PSII	Photosystem II
PAL	Phenylalanine ammonia-lyase
UHPLC-LTQ-IT-MS/MS	Ultra-high-performance liquid chromatography linear trap quadrupole tandem mass Spectrometry
QC	Quality control
MSTFA	*N*-methyl-*N*-trimethylsilyl trifluoroacetamide
ANOVA	Analysis of variance
