# Supplementary material for: Physiological and Metabolomic Responses of Kale to Combined Chilling and UV-A Treatment
Source: Int J Mol Sci. 2019 Oct 8;20(19):4950. doi: 10.3390/ijms20194950 (PMC6801958; doi:10.3390/ijms20194950)
Supplement: Supplementary file 1 [file ijms-20-04950-s001.pdf]

Supplementary Information

Physiological and Metabolomic Responses of Kale to Combined Chilling and UV-A Treatment

Jin-Hui Lee [1,2,†], Min Cheol Kwon [3,†], Eun Sung Jung [4], Choong Hwan Lee [3,4,5] and Myung-Min Oh [1,2,*]

[1] Division of Animal, Horticultural and Food Science, Chungbuk National University, Cheongju 28644, Korea; jhjh@cbnu.ac.kr (J.-H.L); moh@cbnu.ac.kr (M.-M.O)

[2] Brain Korea 21 Center for Bio-Resource Development, Chungbuk National University, Cheongju 28644, Korea

[3] Department of Bioscience and Biotechnology, Konkuk University, Seoul 05029, Korea; alscjf1102@naver.com (M.C.K.); chlee123@konkuk.ac.kr (C.H.L.)

[4] Department of Systems Biotechnology, Konkuk University, Seoul 05029, Korea; jes708@naver.com (E.S.J)

[5] Research Institute for Bioactive-Metabolome Network, Konkuk University, Seoul 05029, Korea

* Correspondence: moh@cbnu.ac.kr; Tel.: +82-43-261-2530

† These authors contributed equally to this work.

Table S1. Differential metabolites identified by UPLC-Q-TOF-MS and UHPLC-LTQ-ESI-IT-MS/MS in kale leaves cultivated under different stress treatments.

NO.	Tentative identification [a]	UPLC-Q-TOF-MS					UHPLC-LTQ-ESI-IT-MS/MS		
		RT(min) [b]	[M-H]$^-$	[M+H]$^+$	Elemental composition	i-FIT (norm)	MS fragement ion (nega)	UV (nm)	ID [c]
Flavonoids									
1	Kaempferol-3-O-triglucoside-7-O-D-diglucoside	3.00	1095.2893	1097.3079	C48H55O29	0.024	1095>933, 771, 609>285	338	Ref[S1]
2	Kaempferol-3-O-hydroxyferuloyl-sophoroside-7-O-D-glucoside	3.00	963.2441	965.2684	C43H47O25	0.042	963>801>609>429, 285	326	Ref[S2]
3	Kaempferol-3-O-caffeoyl-sophoroside-7-O-D-glucoside	3.05	933.233	935.2496	C42H45O24	0.440	933>771>609>429, 285	339	Ref[S2]
4	Kaempferol-3-O-sinapoyl-triglucoside-7-O-D-glucoside	3.13	1139.3213	1141.3250	C50H59O30	0.207	1139>977>771>609>429, 285	268, 333	Ref[S3]
5	Kaempferol-3-O-sinapoyl-sophoroside-7-O-D-glucoside	3.18	977.2589	979.2775	C44H49O25	0.183	977>815>609, 429, 285	245, 268, 331	Ref[S2]
6	Kaempferol 3-O-feruloyl-sophoroside-7-O-D-diglucoside	3.18	1109.3083	1111.3212	C49H57O29	2.139	1109>947, 785>609, 429, 285	268, 332	Ref[S2]
7	Kaempferol-3-O-feruloyl-sophoroside-7-O-D-glucoside	3.23	947.2501	949.2662	C43H47O24	0.061	947>785>623, 609, 591>429, 285	267, 327	Ref[S2]
8	Quercetin-3-O-sophoroside-7-O-D-glucoside	2.73	787.1934	789.2069	C33H39O22	0.048	787>625>301	326	Ref[35]
9	Quercetin-3-O-sinapoyl-sophoroside-7-O-D-glucoside	3.11	993.2529	995.2749	C44H49O26	3.553	993>831>625, 301	338	Ref[S2]
10	Quercetin-3-O-disinapoyl-triglucoside-7-O-D-glucoside	3.79	1361.3898	1363.3556	C65H69O32	1.973	1361>1199, 1155>993>787, 301	268(sh), 332	Ref[S2]
Hydroxycinnamic acids									
11	Caffeoylquinic acid	2.71	353.0852	377.0839 [d]	C16H17O9	n/a	353>191>179	279	Ref[35]
12	5-Feruloyl quinic acid	3.22	367.1014	369.1157	C17H19O9	n/a	367>193, 173>149, 134	269, 328	Ref[S4]
13	1,2-Disinapoylgentiobioside	4.27	753.2252	777.2240 [d]	C34H41O19	0.591	753>529, 289, 223	329	Ref[35]
14	1-Sinapoyl-2-feruloylgentiobioside	4.35	723.2136	747.2121 [d]	C33H39O18	0.192	723>529, 499>259, 193, 175, 160	327	Ref[S5]
15	1,2,2'-Trisinapoylgentiobioside	4.57	959.2864	983.2842 [d]	C45H51O23	0.277	959>735, 529, 511, 427	324	Ref[S5]

[a] Variables were selected based on variable importance of projection (VIP > 0.7) from OPLS-DA (Fig. S2) analyzed by UPLC-Q-TOF-MS
[b] Retention time
[c] Metabolites were identified using reference
[d] Metabolites were detected [M+Na]$^+$ in positive mode

Table S2. Differential metabolites identified by GC-TOF-MS in kale leaves cultivated under different stress treat ments.

NO.	Tentative identification [a]	GC-TOF-MS RT(min) [b]	Identified ion (m/z) [c]	Mass Fragment pattern	TMS [d]	ID [e]
Fatty acids						
16	Linolenic acid	14.2	108	75, 79, 73, 67, 55, 93, 95, 91	1	MS
17	Palmitic acid	13.2	117	73, 75, 117, 132, 129, 55, 145, 45	1	MS
18	Stearic acid	14.4	132	73, 75, 117, 132, 55, 129, 145, 57	1	MS
Amino acids						
19	Threonine	8.4	219	73, 57, 117, 45, 219, 101, 218, 75	3	MS
20	Valine	6.7	218	73, 144, 45, 218, 147, 145, 100, 74	2	MS
21	Phenylalanine	10.4	192	73, 218, 45, 192, 100, 147, 75, 74	2	MS
22	Alanine	5.5	89	116, 73, 147, 117, 45, 59, 148, 66	2	MS
23	Serine	8.1	219	73, 204, 218, 45, 100, 75, 147, 74	3	MS
24	Aspartic acid	8.7	160	73, 160, 75, 130, 45, 117, 116, 74	2	MS
25	GABA	9.6	175	174, 147, 86, 59, 73, 100, 175, 304	3	MS
26	Glutamic acid	10.3	246	73, 246, 75, 45, 128, 84, 147, 156	3	MS
27	Lycine	12.5	317	174, 156, 59, 128, 86, 100, 230, 317	4	MS
28	Isoleucine	7.5	158	158, 73, 218, 45, 100, 159, 59, 69	2	MS
29	Proline	7.5	142	142, 73, 45, 75, 143, 74, 66, 70	2	MS
30	Glycine	7.6	174	174, 86, 100, 175, 248, 176, 249, 276	3	MS
Organic acids						
31	Pyruvic acid	5.1	174	73, 174, 45, 74, 59, 89, 115, 72	1	MS
32	Succinic acid	7.6	247	147, 73, 75, 45, 55, 148, 56, 149	2	MS
33	Propanoic acid	7.8	189	73, 147, 189, 45, 103, 102, 133, 75	3	MS
34	Citric acid	11.8	211	73, 147, 45, 67, 273, 211, 133, 183	4	MS
35	Lactic acid	5.2	117	73, 117, 147, 45, 66, 75, 59, 74	2	MS
36	Benzoic acid	7.1	179	105, 179, 77, 135, 51, 180, 45, 50	1	MS
37	Maleic acid	7.6	245	73, 147, 45, 148, 75, 245, 66, 149	2	MS
38	Fumaric acid	7.9	245	245, 73, 75, 45, 143, 47, 115, 147	2	MS
39	Malic acid	9.1	323	73, 147, 45, 55, 75, 74, 133, 233	3	MS
40	Quinic acid	12.1	345	73, 345, 147, 255, 75, 45, 133, 346	5	MS
Sugar & Sugar derivatives						
41	myo-Inositol	13.7	191	73, 147, 217, 191, 45, 129, 74, 103	6	MS
42	Xylitol	10.9	319	73, 103, 147, 45, 217, 129, 74, 75	5	MS
43	Sucrose	16.7	361	73, 103, 147, 217, 361, 129, 74, 75	8	MS
44	Gluconic acid	13.1	333	73, 147, 103, 74, 45, 75, 292, 333	5	MS
45	Threonic acid	9.8	292	73, 147, 45, 117, 103, 74, 292, 220	4	MS
46	Xylose	10.8	103	73, 103, 217, 147, 45, 74, 59, 75	4	MS
47	Fructose	12.3	196	73, 103, 147, 217, 45, 89, 74, 59	5	MS
48	Glucose	12.4	272	103, 117, 160, 129, 89, 133, 59, 157	5	MS
49	Galactose	12.6	160	73, 103, 147, 160, 45, 117, 133, 129	5	MS
50	Maltose	17.3	361	73, 147, 204, 103, 45, 217, 361, 74	8	MS
Hydroxycinnamic acids						

51	Caffeic acid	13.8	219	73, 219, 45, 75, 396, 191, 220, 59	3	MS
52	Sinapic acid	14.4	338	73, 45, 338, 75, 368, 59, 353, 339	2	MS
53	Ferulic acid	13.5	338	73, 45, 75, 338, 249, 308, 323, 59	2	MS

[a] Tentative different metabolites identified by mass spectrum consistent with those of standard compound, NIST, and in-house library and the different metabolites based on variable projection (VIP > 0.7) from OPLS-DA (Fig. S2) analyzed by GC-TOF-MS

[b] Retention time

[c] The selected ion is m/z value for identification and quantification

[d] TMS : trimethylsilyl

[e] Identification : MS, mass fragment pattern of NIST and in-house library

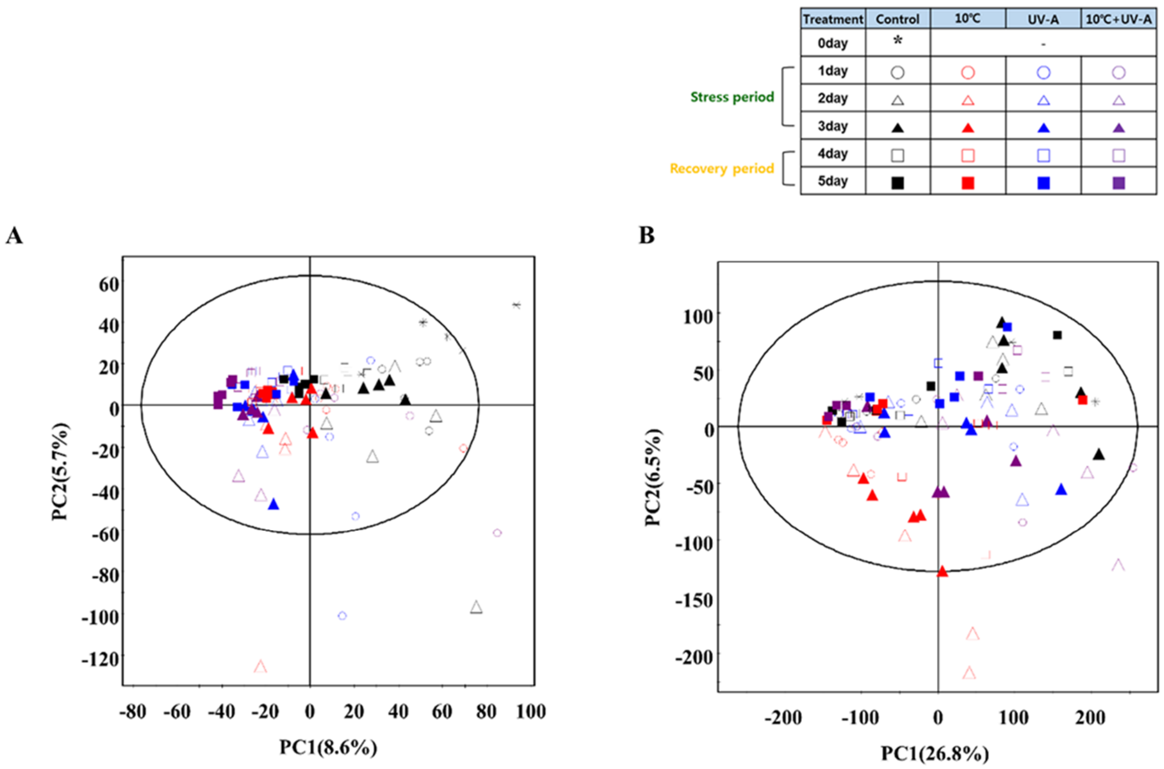

Figure S1. Principal component analysis (PCA) score plots derived from UPLC-Q-TOF-MS (**A**) and GC-TOF-MS (**B**) data for kale leaves cultivated under 10°C, UV-A and 10°C+UV-A.

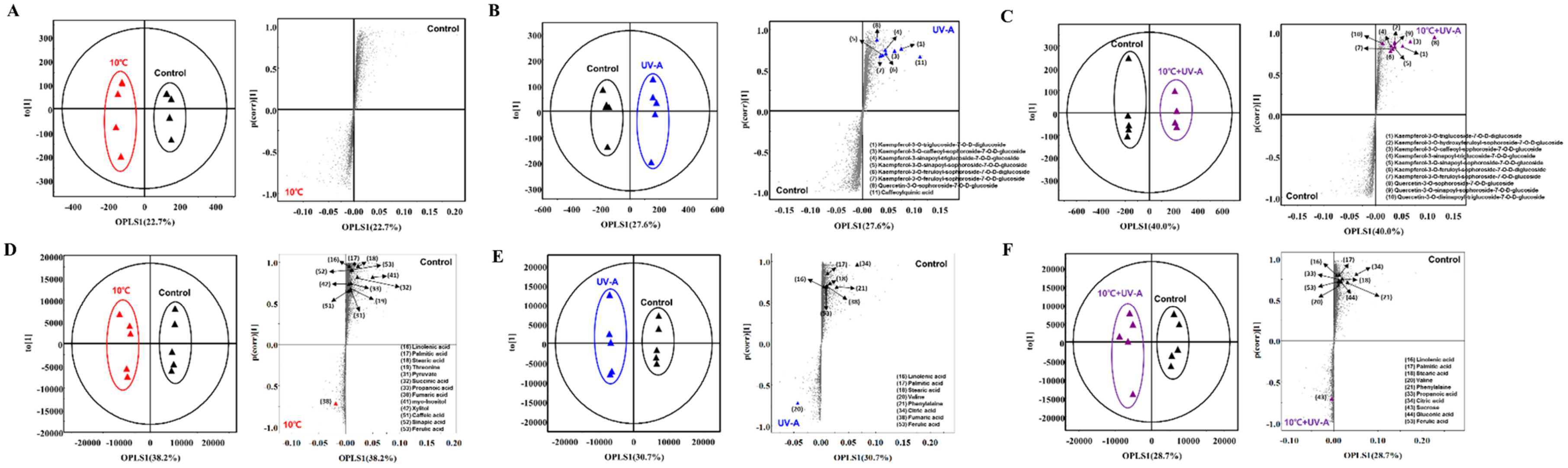

Figure S2. Orthogonal partial least squares-discriminant analysis (OPLS-DA) score plots and corresponding loading-S plot derived from UPLC-Q-TOF-MS (**A, B, C**) and GC-TOF-MS (**D, E, F**) data for kale leaves cultivated under 10°C, UV-A and 10°C+UV-A at 3 days. In the loading S-plot based on OPLS-DA data sets, each discriminant metabolites is selected at VIP > 0.7 and *p*-value < 0.05. (**A, D**) Control vs 10°C; (**B, E**) Control vs UV-A; (**C, F**) Control vs 10°C+UV-A.

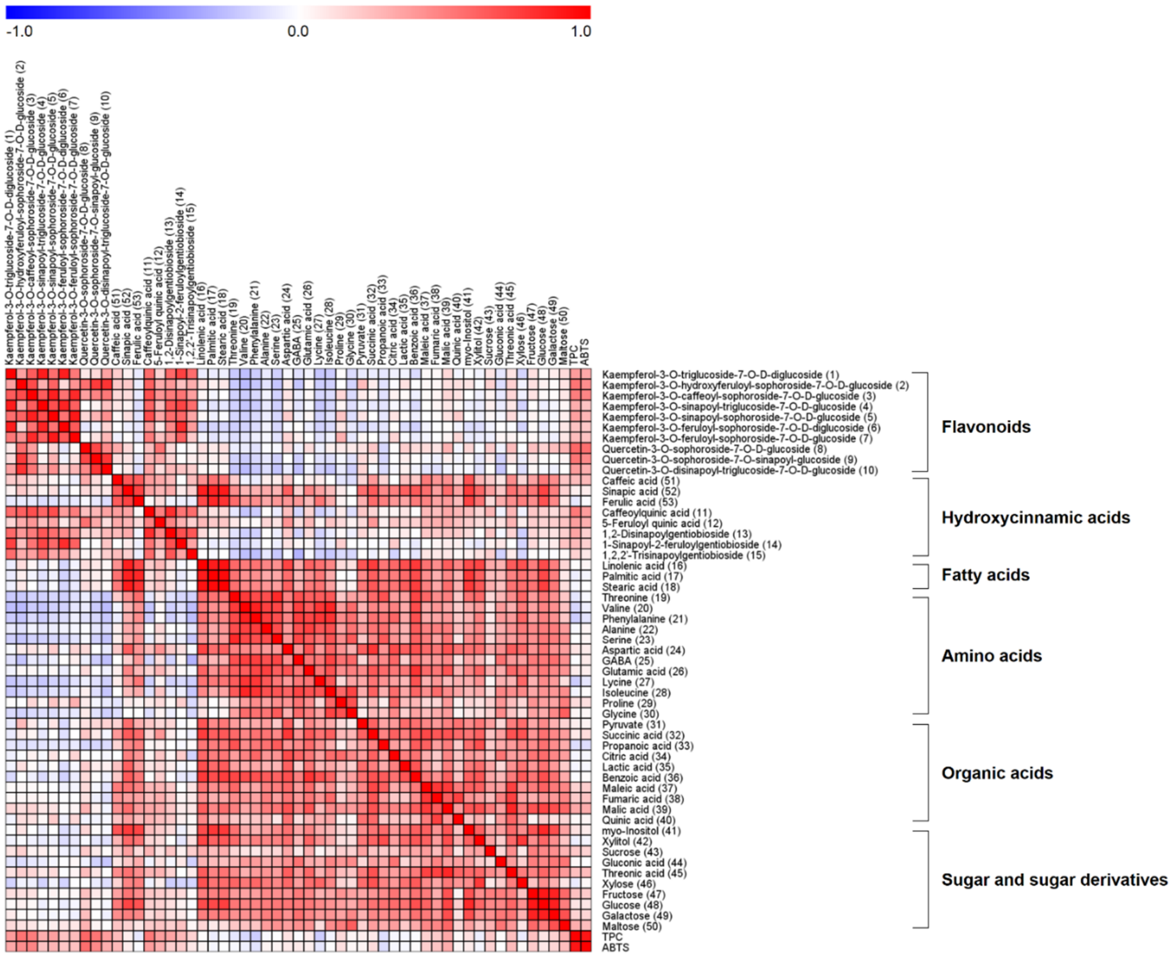

Figure S3. Correlation map between the metabolite levels and observed bioactivites (total phenolic content and antioxidant capacity). Each metabolite is identified as significantly different metabolites through OPLS-DA (Figure S2). Each square indicates Pearson's correlation coefficient of a pair of metabolites and assayed activities. The red color indicates a positive (0 < r <1) correlation and the blue colors indicates negative (-1 < r < 0) correlation.

Supplementary references

1. Ferreres, F.; Fernandes, F.; Oliveira, J. M.; Valentão, P.; Pereira, J. A.; Andrade, P. B. Metabolic profiling and biological capacity of Pieris brassicae fed with kale (*Brassica oleracea* L. *var. acephala*). *Food Chem. Toxicol.* **2009**, *47*, 1209-1220. doi: 10.1016/j.fct.2009.02.014
2. Schmidt, S.; Zietz, M.; Schreiner, M.; Rohn, S.; Kroh, L. W.; Krumbein, A. Identification of complex, naturally occurring flavonoid glycosides in kale (*Brassica oleracea* var. *sabellica*) by high-performance liquid chromatography diode-array detection/electrospray ionization multi-stage mass spectrometry. *Rapid Commun. Mass Spectrom.* **2010**, *24*, 2009-2022. doi: 10.1002/rcm.4605
3. Llorach, R.; Gil-Izquierdo, A.; Ferreres, F.; Tomás-Barberán, F. A. HPLC-DAD-MS/MS ESI characterization of unusual highly glycosylated acylated flavonoids from cauliflower (*Brassica oleracea* L. *var. botrytis*) agroindustrial byproducts. *J. Agric. Food Chem.* **2003**, *51*, 3895-3899. doi: org/10.1021/jf030077h
4. Xiao, Y. C.; Liu, L. T.; Bian, J. J.; Yan, C. Q.; Ye, L.; Zhao, M. X.; Huang, Q. S.; Wang, W.; Liang, K.; Shi, Z. F.; Ke, X. Identification of multiple constituents in shuganjieyu capsule and rat plasma after oral administration by ultra-performance liquid chromatography coupled with electrospray ionization and ion trap mass spectrometry. *Acta Chromatogr.* **2018**, *30*, 95-102. doi: org/10.1556/1326.2017.00094
5. Velasco, P.; Francisco, M.; Moreno, D. A.; Ferreres, F.; García-Viguera, C.; Cartea, M. E. Phytochemical fingerprinting of vegetable *Brassica oleracea* and *Brassica napus* by simultaneous identification of glucosinolates and phenolics. *Phytochem. Anal.* **2011**, *22*, 144-152. doi.org/10.1002/pca.1259
